# Implementation and evaluation of a multi-level mental health promotion intervention for the workplace (MENTUPP): study protocol for a cluster randomised controlled trial

**DOI:** 10.1186/s13063-023-07537-0

**Published:** 2023-09-30

**Authors:** Ella Arensman, Mallorie Leduc, Cliodhna O’Brien, Paul Corcoran, Eve Griffin, Caleb Leduc, Evelien Coppens, Fotini Tsantila, Victoria Ross, Kahar Abdulla, Pia Hauck, Benedikt L. Amann, Birgit Aust, Arlinda Cerga Pashoja, Johanna Cresswell-Smith, Luigia D’Alessandro, Naim Fanaj, Birgit A. Greiner, Jeroen Luyten, Sharna Mathieu, Margaret Maxwell, Gentiana Qirjako, Hanna Reich, Sarita Sanches, Monika Ditta Tóth, Joseph Kilroy, Karen Michell, Nicola Reavley, David McDaid, Chantal Van Audenhove, the MENTUPP consortium, Ainslie O’Connor, Ana Moreno-Alcázar, Andia Meksi, Andras Szekely, Anthony LaMontagne, Ariel Como, Arilda Dushaj, Asmae Doukani, Azucena Justicia, Bridget Hogg, Carolyn Holland, Charlotte Paterson, Chris Lockwood, Daniel Guinart, Doireann Ní Dhálaigh, Dooyoung Kim, Eileen Williamson, Eva Zsak, Genc Burazeri, Grace Cully, Grace Davey, György Purebl, Ilinca Serbanescu, Jaap van Weeghel, Juan Carlos Medina Alcaraz, Joe Eustace, Juliane Hug, Kairi Kõlves, Karen Mulcahy, Katherine Thomson, Kristian Wahlbeck, Lars de Winter, Laura Cox, Marta Fontana, Peter Trembeczky, Pia Driessen, Rebecca Lohmann-Devantier, Reiner Rugulies, Ruth Benson, Saara Rapeli, Sarah Ihinonvien, Sevim Mustafa, Stefan Hackel, Susan Alexander, Tanya King, Ulrich Hegerl, Vanda Scott, Wendy Orchard

**Affiliations:** 1 School of Public Health, University College Cork, Cork, Ireland; 2 National Suicide Research Foundation, Cork, Ireland; 3 Australian Institute for Suicide Research and Prevention, School of Applied Psychology, Griffith University, Mount Gravatt, QLD Australia; 4 Centre for Care Research and Consultancy, LUCAS, KU Leuven, Louvain, Belgium; 5 European Alliance Against Depression E.V, Leipzig, Germany; 6 Centre Fòrum Research Unit, Institut de Neuropsiquiatria I Addiccions, Barcelona, 08019 Spain; 7 Hospital del Mar Medical Research Institute (IMIM), Barcelona, Spain; 8 Centro de Investigación Biomédica en Red de Salud Mental (CIBERSAM), Instituto Carlos III, Madrid, Spain; 9 Univ. Pompeu Fabra, Barcelona, Spain; 10 The National Research Centre for the Working Environment, Copenhagen, Denmark; 11 London School of Hygiene and Tropical Medicine, London, UK; 12 Finnish Institute for Health and Welfare (THL), Helsinki, Finland; 13 International Association for Suicide Prevention (IASP), Washington, DC USA; 14 College of Medical Sciences Rezonanca, Mental Health Center Prizren, Prishtina, Kosovo; 15 Department of Public Health and Primary Care, Faculty of Medicine, Leuven Institute for Healthcare Policy, KU Leuven, Belgium; 16 Nursing, Midwifery and Allied Health Professions Research Unit (NMAHP-RU), Faculty of Health Sciences and Sport, University of Stirling, Stirling, UK; 17 Department of Public Health, Faculty of Medicine, University of Medicine, Tirana, Albania; 18 German Depression Foundation, Leipzig, Germany; 19 Department of Psychiatry, Psychosomatic Medicine and Psychotherapy, Depression Research Centre of the German Depression Foundation, University Hospital, Goethe University, Frankfurt Am Main, Germany; 20 Phrenos Center of Expertise for Severe Mental Illness, Utrecht, The Netherlands; 21 Altrecht Mental Health Care, Utrecht, The Netherlands; 22 Institute of Behavioural Sciences, Semmelweis University, Budapest, Hungary; 23 The Chartered Institute of Building, Dublin, Ireland; 24 Institution of Occupational Safety and Health, Leicester, UK; 25 School of Population and Global Health, Faculty of Medicine, Dentistry and Health Sciences, Centre for Mental Health, The University of Melbourne, Melbourne, Australia; 26 Care Policy and Evaluation Centre, Department of Health Policy, London School of Economics and Political Science, London, UK; 27 Academic Center for General Practice, KU Leuven, Louvain, Belgium

**Keywords:** Depression, Mental health and wellbeing, Occupational, Organisational interventions, Process evaluation, Economic evaluation, Self-harm, Suicidal behaviour, Suicide, Workplace health promotion, Workplace-based health interventions, Implementation

## Abstract

**Background:**

Well-organised and managed workplaces can be a source of wellbeing. The construction, healthcare and information and communication technology sectors are characterised by work-related stressors (e.g. high workloads, tight deadlines) which are associated with poorer mental health and wellbeing. The MENTUPP intervention is a flexibly delivered, multi-level approach to supporting small- and medium-sized enterprises (SMEs) in creating mentally healthy workplaces. The online intervention is tailored to each sector and designed to support employees and leaders dealing with mental health difficulties (e.g. stress), clinical level anxiety and depression, and combatting mental health-related stigma. This paper presents the protocol for the cluster randomised controlled trial (cRCT) of the MENTUPP intervention in eight European countries and Australia.

**Methods:**

Each intervention country will aim to recruit at least two SMEs in each of the three sectors. The design of the cRCT is based on the experiences of a pilot study and guided by a Theory of Change process that describes how the intervention is assumed to work. SMEs will be randomly assigned to the intervention or control conditions. The aim of the cRCT is to assess whether the MENTUPP intervention is effective in improving mental health and wellbeing (primary outcome) and reducing stigma, depression and suicidal behaviour (secondary outcome) in employees. The study will also involve a process and economic evaluation.

**Conclusions:**

At present, there is no known multi-level, tailored, flexible and accessible workplace-based intervention for the prevention of non-clinical and clinical symptoms of depression, anxiety and burnout, and the promotion of mental wellbeing. The results of this study will provide a comprehensive overview of the implementation and effectiveness of such an intervention in a variety of contexts, languages and cultures leading to the overall goal of delivering an evidence-based intervention for mental health in the workplace.

**Trial registration:**

Please refer to Item 2a and registration ISRCTN14104664. Registered on 12th July 2022.

**Supplementary Information:**

The online version contains supplementary material available at 10.1186/s13063-023-07537-0.

## Background

Mental health difficulties and disorders in the workplace severely impact businesses through absenteeism/presenteeism, decreased productivity, workplace accidents and even self-harm and suicide [1]. Employees in some sectors may be more at risk of experiencing mental health difficulties (e.g. burnout) or disorders, such as depression and anxiety. Male-dominated workplaces, such as the construction and ICT sectors, are characterised by high levels of mental health-related stigma and low levels of help-seeking for mental health concerns, despite increased psychological distress [2]. Paradoxically, stigmatising attitudes are common in healthcare professionals, which can have a negative impact on self-care behaviours [3]. These sectors have also been associated with an increased risk of suicide among workers [4], with male construction workers in the UK being almost four times more likely to die by suicide than the general working population [5].

Certain workplace-related factors may contribute to the development, exacerbation and maintenance of mental health difficulties. For example, an association with the risk for depressive disorders has been found for the combination of high demands and low control [6], for the imbalance between high efforts and low rewards [7] and for workplace bullying [8], making the workplace an important setting for interventions. Organisation size is another factor which may contribute to employee wellbeing. Small- to medium-sized enterprises (SMEs) are the most common form of workplace in the European Union and they employ a significantly larger share of employees compared to larger organisations [9]. Research indicates that SMEs face additional workplace stressors, such as social isolation, long work hours, out-dated technologies, lack of human resource management systems, economic uncertainty and financial pressures [10, 11]. These are exacerbated by a lack of infrastructure to develop expertise, knowledge and resources to invest in workplace occupational health programmes [12–14]. Although SMEs may not usually have the financial resources to address employee mental health, they may be in a unique position to promote mental health, prevent clinical and non-clinical mental health difficulties and reduce mental health-related stigma. The COVID-19 pandemic has further exacerbated risk factors contributing to mental health difficulties while greatly reducing social interactions and opportunities for support from colleagues. It is anticipated that the long-lasting impacts of the pandemic may include increased levels of depression, self-harm and suicidality [15–17] as well as anxiety, post-traumatic stress disorder and sleep disorders, which may be compounded by job insecurity and long periods of isolation associated with the pandemic [18]. Europe, in particular, now is faced with a new economic crisis, caused by the war in Ukraine and the rapid increase in energy prices that are also placing great financial worries on both business and the general public.

According to a systematic meta-review, there is moderate evidence that workplace factors such as high job demands, low job control, role stress, bullying and low social support can be associated with a heightened risk for the onset of negative mental health outcomes [19]. Workplaces that intervene to offset these factors are more likely to reduce negative mental health outcomes, as well as absenteeism and presenteeism, and ultimately, may increase productivity and contribute to economic gains [11, 20–22]. Research in the construction sector has shown that the implementation of an occupational intervention for minor depressive symptoms may prevent more serious depressive symptoms, including suicidal thoughts and behaviours [23, 24]. Furthermore, some evidence suggests educational interventions in the healthcare sector are effective in decreasing mental health-related stigma [3, 25].

The literature on workplace mental health interventions indicates that a multi-level approach which encompasses the organisational level and the individual level can be successful in reducing burnout [26–29], anxiety [30], stress [31, 32] and stigma [33]. A key factor in the multi-level approach is the active role that management needs to play in ensuring the intervention fits with existing policies and practices within the organisation as well as providing the resources for implementation [34, 35]. Management has the ability to directly influence workplace stressors through policy, work redesign or modification of work processes and support structures [36]. Another key factor is the implementation of components that directly target the individual within the organisation, thereby implementing a top-down (via management) and a bottom-up (via employees) approach [37]. Successful workplace mental health interventions also incorporate members of the workforce in their planning and delivery, reinforcing the value of both organisational and individual level buy-in [38].

In addition to a multi-level approach, interventions that are tailored to the needs of the specific workplace or sector have the potential to be more effective [39]. This includes presenting information using images and scenarios which the individual would find relatable and providing a variety of intervention components that workplaces can choose from to fit the intervention activities to their actual needs [40]. Accessible workplace interventions are also integral to effective implementation. In recent years, interventions delivered through digital technologies have been successful in engaging a wide range of users across the organisation [41] and in reducing stress [42] and mild depressive symptoms [43].

Well-organised and managed workplaces can be a source of wellbeing [44–46]. There has been an increase in workplaces interested in supporting their employees to prevent, detect and manage mental health difficulties and disorders in the workplace [14]. However, only 7% of mental health promotion and prevention programmes globally are workplace-based [47]. There is a need and demand for methodologically sound research studies to examine the efficacy of implementing mental health interventions designed for the workplace. At present, there is no known multi-level, tailored, flexible and accessible workplace-based mental health intervention targeting a spectrum of non-clinical and clinical symptoms. The EU Horizon 2020 project Mental Health Promotion and Intervention in Occupational Settings (MENTUPP) aims to fill this gap.

The MENTUPP workplace intervention targets clinical mental disorders (depression and anxiety disorders), non-clinical mental health problems (stress, burnout, depressive symptoms) and mental health stigma and supports positive mental wellbeing by adopting the multi-level approach. The MENTUPP intervention aims to protect mental health by promoting mental health in the workplace (primary prevention), reducing work-related risk factors (secondary prevention) and targeting mental health problems as they develop (tertiary prevention) [48] by providing guidance and support for all employees and management in the organisation. The MENTUPP approach adopts several theoretical frameworks including the Integrated Mental Health Framework by LaMontagne and colleagues [49] which includes three threads: (i) Protect mental health by controlling harmful work exposures; (ii) promote mental health by building on strengths and (iii) address mental health problems regardless of cause. Furthermore, it uses the Dual Mental Dual Continuum Wellbeing Framework by Keyes to differentiate between mental health and mental wellbeing [50]. The materials developed for the MENTUPP intervention are tailored to three at-risk sectors: construction, healthcare and information and communication technology (ICT) to enhance the uptake and potential effectiveness [51] and are available online through the MENTUPP Hub. Digital interventions are appropriate for increasing reach [52], particularly in the context of sectors that have inflexible shift patterns (i.e. healthcare, ICT), that have employees working on various sites (i.e. construction) or employees working remotely (ICT).

The overall goal of the MENTUPP intervention is to improve mental health and mental wellbeing in the workplace, with secondary aims of reducing stigma, depression and suicidal behaviour by providing tailored mental health promotion materials for the construction, healthcare and ICT sectors. MENTUPP provides SMEs with easily accessible tools for leaders and for employees that make it possible to address mental health issues on the workplace-level and the employee level.

## Objectives

Based on the results and experiences of a pilot study [53, 54], the present paper outlines the protocol for the cluster Randomised Controlled Trial (cRCT) which will investigate the effectiveness of the intervention in improving mental health and wellbeing (primary outcome) and reducing stigma, depression and suicidal behaviour (secondary outcome) in employees. Furthermore, multi-level workplace interventions always interact within context and can not only be assessed for their effects on mental health outcomes, but need to also have a sufficient process evaluation in order to investigate the mechanisms of effectiveness [55]. Given that resources are limited it is also insufficient just to look at the effectiveness of interventions, it is important to also consider their relative cost-effectiveness. This is particularly important in the case of SME’s which can be operating under conditions of substantial financial uncertainty. Therefore, the study will also involve a process and economic evaluation. In this article, we will present the protocol for the cRCT of the MENTUPP intervention in eight European countries and Australia.

## Methods/design

The manuscript has been developed in line with the SPIRIT reporting guidelines [56].

### Study design

**Fig. 1:**
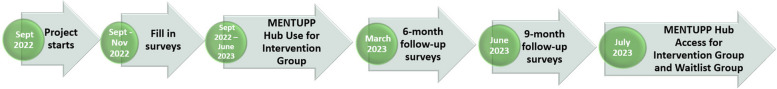
MENTUPP cRCT timeline

A cRCT will be conducted with SMEs in two conditions, intervention and control, within each of the three sectors: healthcare, construction and ICT. Quantitative and qualitative evaluation data will be obtained and guided by the RE-AIM framework [57] and the evaluation framework of Proctor et al. [58], to structure the outcome, process and economic evaluation of the MENTUPP intervention from data collected at baseline, 6-month and 9-month follow-ups. Five types of evaluation measures will be collected: six validated questionnaires, two self-developed surveys, a monitoring instrument and user data from the MENTUPP Hub. Focus groups conducted with the local research officers post-intervention will inform process evaluation and how the Hub was implemented locally. The intervention group will complete the baseline survey and receive immediate access to the MENTUPP Hub. They will also complete the 6-month and 9-month follow-up measures. The control group will complete the baseline, 6-month and 9-month measures. Following the completion of the 9-month measures, participants of the control group will receive access to the MENTUPP Hub (see Fig. 1 for timeline).

### Study population

#### Organisations

Organisations that fit the criteria of being an SME will be invited to participate in the study. SMEs are defined as organisations with fewer than 250 employees [9]. A small sized enterprise is defined as an organisation with fewer than 50 employees and a medium-sized enterprise consists of 50–249 employees. Definitions and inclusion criteria of the SMEs in the construction, healthcare and ICT sectors are based on Nomenclature of Economic Activities (NACE) guidelines [59]. The aim is to recruit a minimum of two SMEs from each of the three targeted sectors, construction, healthcare and ICT, across the nine participating countries: Australia, Albania, Ireland, Netherlands, Hungary, Kosovo, Germany, Finland and Spain. If significant issues arise with the recruitment of SMEs as per the strict criteria, consideration will be given to broaden the definition to include slightly larger organisations (e.g. 300 employees).

### Participants

Individuals will be eligible to participate in the MENTUPP intervention if they are (1) full- or part-time employees, including contractors, supervisors and individuals on sick leave or other types of authorised leave (e.g. maternity or care leave); (2) employed within an eligible SME in the construction, healthcare or ICT sector; (3) aged 18 years or older; and (4) willing to give their informed consent for participation. Any individual within an organisation who has any type of managerial or leadership role will be referred to as ‘leader’ in the MENTUPP project.

### Sample size

In each of the nine countries, local research officers will use convenience sampling to recruit a minimum of two SMEs in each of the three sectors, yielding a total of at least 54 participating SMEs, half for intervention and half for control. An average of 65 participants per SME will be recruited at baseline. Allowing for an estimated attrition rate of 65%, based on the pilot study and previous literature [60, 61], this will yield baseline and follow-up data for 23 participants per SME. A sample size of 621 participants (i.e. 27 SMEs with 23 participants) in each arm of the cRCT achieves 85% power to detect a difference of 0.25 standard deviations between the mean change in intervention participants and the mean change in control participants, when the intra-cluster correlation is 0.05, using a two-sided independent *t*-test with a significance level of 0.05.

### Description of the study intervention

#### Materials

**Fig. 2:**
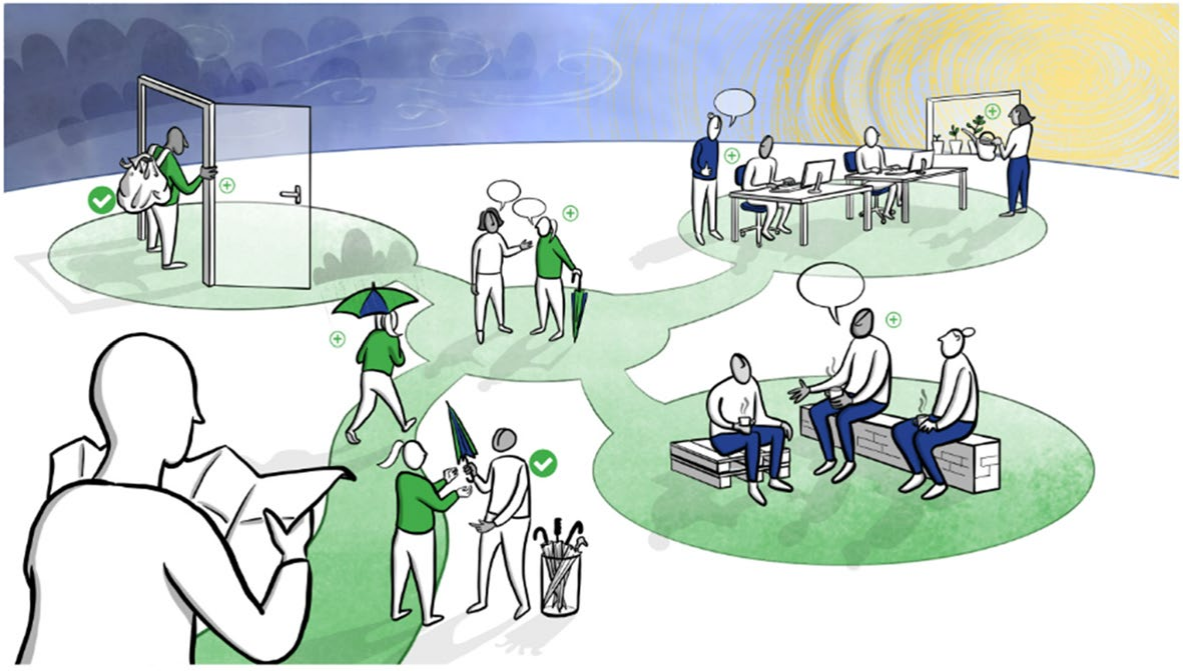
Screenshot of MENTUPP Hub springboard page which links to modules

The MENTUPP intervention has been developed and designed to improve mental health and wellbeing and reduce stigma in the workplace and is facilitated through the MENTUPP Hub, an online platform that presents interactive psychoeducational materials, toolkits and links to additional resources (See Fig. 2—screenshot of Hub).

**Fig. 3:**
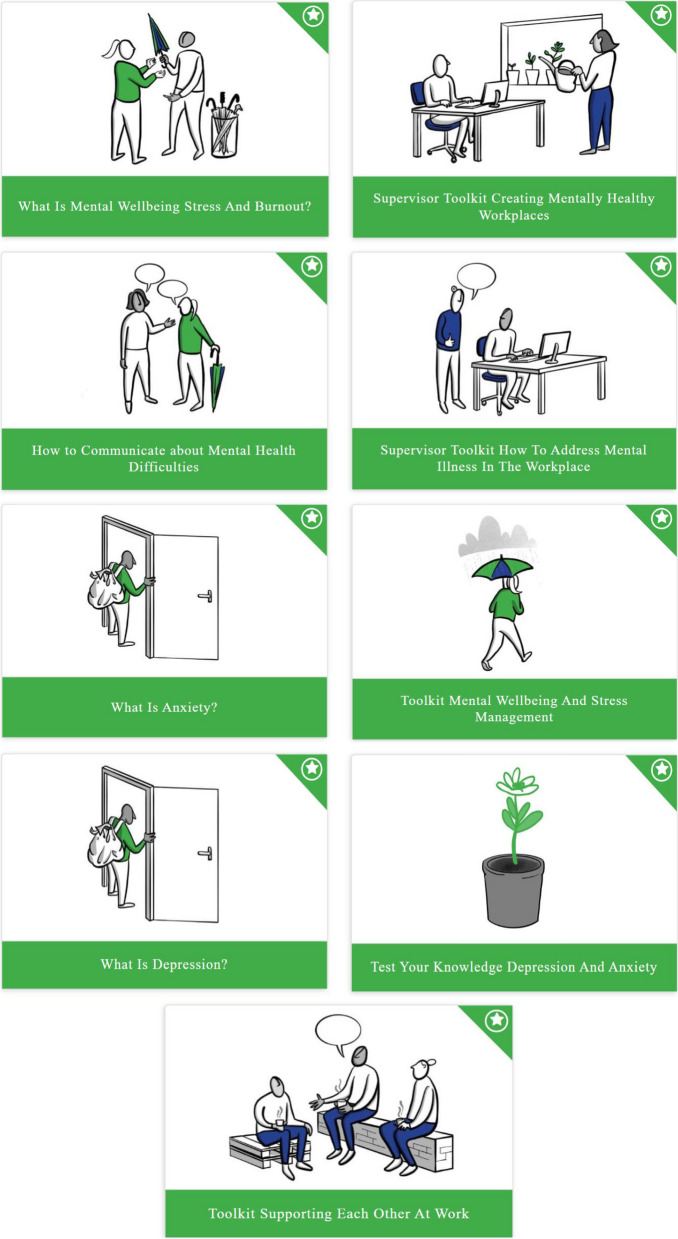
Screenshot of module links within MENTUPP Hub

All online intervention materials designed and developed for the MENTUPP Hub were optimised following a pilot study in relation to the key messages, content, visuals and the structure [53]. The optimisation process was informed by a series of systematic reviews on the implementation of mental health promotion interventions in workplace settings and on psychosocial interventions for depression, anxiety and suicidal ideation and behaviours in SMEs [62–64], recommendations from local research officers received during the pilot study, the results of the evaluation measures and focus groups from the pilot study, and feedback from local Steering Groups comprised of representatives from each of the sectors. The main changes to the online intervention materials were informed by a synthesis of the collated documents to produce a more engaging, coherent, user-friendly experience with simple language and improved structure and navigation throughout the online platform (see Figs. 2 and 3).

The optimised MENTUPP Hub is comprised of eight modules with tailored visuals, videos and case study content within each sector, which are also tailored to different organisations levels. MENTUPP is a multi-level intervention designed to target all levels of the organisation; the employees, management and the organisation as a whole. Furthermore, materials are tailored for each sector to ensure that the language and content of materials is relevant to the construction, healthcare and ICT sectors. The interactive materials are delivered through online information packages, videos, pre-recorded roleplays, animated scenarios of case studies, short quizzes, reflection exercises, breathing and mindfulness exercises and practical stress management exercises [53].

### Languages

All materials in the MENTUPP Hub and the associated evaluation will be available in the national languages spoken in the intervention countries: Albanian, Dutch, English, Finnish, German, Hungarian and Spanish. Three additional languages, Polish, Turkish and Ukrainian, that are frequently spoken among employees in the recruited SMEs determined by the pilot and local steering groups, will also be available. The language of the informed consent and registration process is dictated by the country location of initial access. Following informed consent and registration, participants may select their preferred language to engage with the MENTUPP Hub.

### Procedure

#### SME recruitment

The standard operating procedures utilised in the pilot study will also be used to guide the recruitment and initial interaction and engagement with SMEs for the local research officers to assess eligibility, participation and commitment [53]. In addition to seeking guidance on recruitment of SMEs from local steering groups, SMEs who participated in the pilot study will also be contacted for snowball recruitment.

### Randomisation of SMEs

Local research officers will assign a pseudonym to each participating SME. SMEs will be allocated to either the intervention or control conditions using block randomisation. This will be completed by the evaluation team, who remain blinded to the identity of the SMEs. To maintain balance between the intervention and control groups, group allocation will be conducted in pairs of SMEs within each sector in each country. This process will be repeated to create a list of SMEs with group allocation. Minimisation is expected to be used when an unpaired SME is required to be allocated to a condition [65]. The first SME will be allocated to the intervention or control group at random. The allocation status of the subsequent SME will be determined based on the allocation of the first SME in a manner that would lead to better balance between the groups in the variables of interest.

After randomisation is complete, the local research officers will contact each SME to inform them of their randomisation status. The meeting will involve discussing the appointment of the designated communications person within the SME, the purpose of the surveys and what is involved in them and clarifying the details of implementing and scheduling the introductory sessions within the SME. Participants and the local research officer will not be blinded to allocation status; however, analysts evaluating the outcome will be blinded.

### Individual recruitment

In partnership with the SME, the local research officers will conduct introductory sessions with the employees including a short presentation describing the purpose of the research. The local research officers will emphasise that participation is voluntary, and employers will not be notified about who is participating within their organisation. Participants will also be informed that they can withdraw from the research at any time without repercussions. Participants will also be given the opportunity to ask any questions they may have. The research officer will provide information on who to contact and how to contact them for any support or assistance. All employees will receive a link to the information sheet and an informed consent form.

For the intervention group, the local research officer will also explain the registration process for access to the MENTUPP Hub and what materials are available on the Hub. For the control group, the local research officer will explain that they will be given access to the MENTUPP Hub at the end of the study period.

### Implementation

The MENTUPP Hub is designed to be accessed by employees and their leaders who have completed the baseline questionnaires on a personal or work electronic device. Given the multi-level approach underpinning the intervention, the MENTUPP project is dependent on achieving buy-in from management within each SME. SMEs are advised to allow participants the opportunity to access and engage with the MENTUPP Hub during work hours, on average 20 min per week throughout the project. Participants can also access the Hub at any time and engage with the material at their own pace during their free time. To promote the intervention within each organisation, SMEs will be encouraged to designate a ‘Champion’ within their organisation. The local research officer will work with the ‘Champion’ by following a standardised method in the form of a series of checklists to encourage discussion and to determine the best approach to implementing the intervention within each organisation. This will ensure that all organisations have flexibility to implement the intervention in the most suitable way for them while still adopting a standardised approach across the nine intervention countries. The ‘Champion’ will determine the best methods for promoting engagement within the organisation and will be involved in boosting participation and retention at all levels. They will disseminate information from research officers to all employees and leaders within the organisation and provide feedback on any challenges or queries to research officers. Standardised information will include email templates and SMS text reminders to access the MENTUPP Hub and complete follow-up surveys. SMEs and participants within SMEs can withdraw their participation at any point of the study without repercussions. Participants can withdraw their data by contacting their local research officer and referring to their unique code.

If any changes are required to the recruitment, implementation or evaluation procedure due to unforeseen circumstances, this will be discussed at weekly implementation meetings with the local research officers. Any modifications required will be noted in the Standard Operating Procedure. Any major modifications will be noted to the funding agency.

### Data collection

**Fig. 4:**
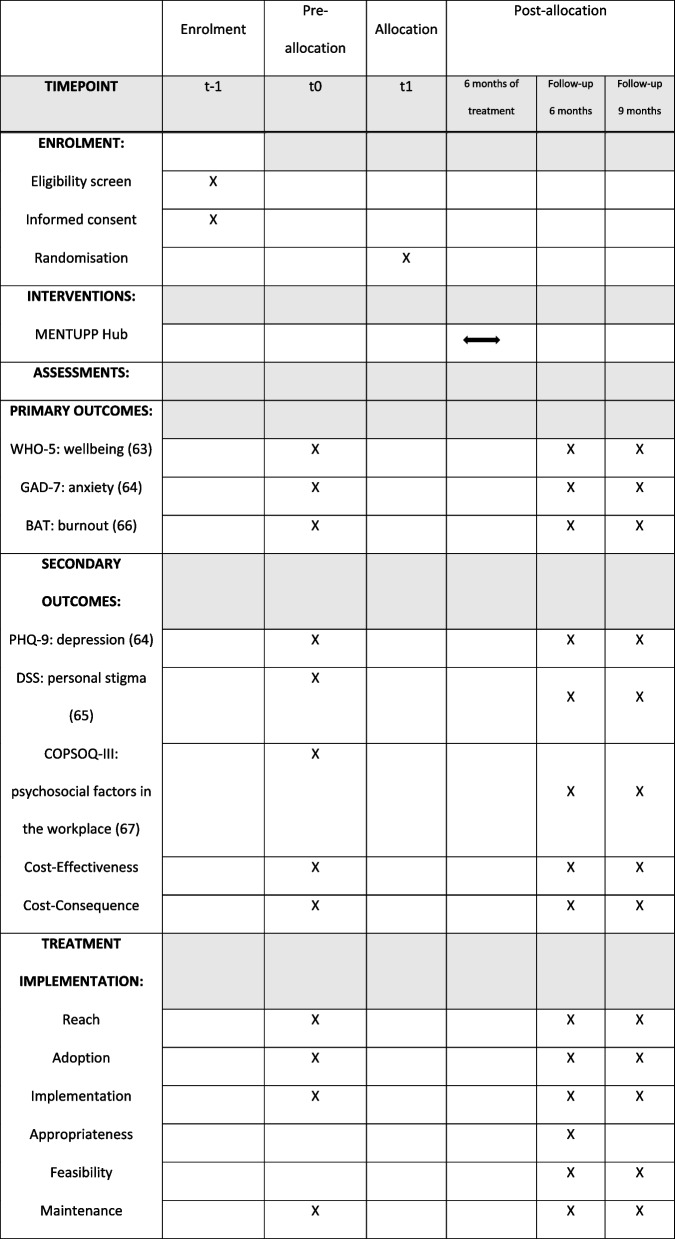
SPIRIT figure of MENTUPP study protocol

Quantitative and qualitative measures will be utilised to collect all necessary data for outcome evaluation, process evaluation, and economic evaluation of the MENTUPP intervention. Measures were selected based on the Theory of Change (ToC) expected as a result of the intervention [66]. An overview of assessment is presented in Fig. 4 and further detailed information is presented in Table 1.

**Table 1:** Overview of measures, evaluation and outcomes

Type of evaluation			Concept	Type of outcomes / perspective	Outcome	Measure											Time of data collection		
**Outcome**	**Proce** **ss**	**Economic**				**Validated scales**						**Bespoke survey**							
						**WHO**	**PHQ-9**	**GAD-7**	**BAT**	**DSS**	**COPSOQ**	**Pre**	**Post**	**Monitoring**	**User data**	**Focus groups**	**Baseline**	**6 months**	**9 months**
X		X	Effectiveness	Long-term Outcomes	Improved wellbeing	X											X	X	X
X		X			Reduced mental illness: depression, anxiety, and burnout		X	X	X								X	X	X
X		X			Reduced personal stigma towards mental illness					X							X	X	X
X					Reduced productivity loss							X	X				X	X	X
		X			Support provided to employees facing mental health difficulties							X	X				X	X	X
		X			Attitudes to search for psychological support							X	X				X	X	X
X				Intermediate Outcomes	Improved psychosocial factors in the workplace						X						X	X	X
X					Improved support of employees facing mental health difficulties						X	X	X				X	X	X
X					More inclusive atmosphere promoting positive psychosocial factors							X	X				X	X	X
X					Improved attitudes to search for psychological support							X	X				X	X	X
X				Proximate Outcomes	Employees and leaders have increased knowledge on mental wellbeing, mental illness and stigma							X	X				X	X	X
X					Employees and leaders have enhanced skills to promote mental health, deal with mental illness and prevent stigma							X	X				X	X	X
X					Employees and leaders have more positive attitudes towards mental illness and help-seeking							X	X				X	X	X
X					Leaders have enhanced skills to address mental illness and prevent mental harm							X	X				X	X	X
X	X				The MENTUPP intervention is implemented as intended									X			X	X	X
	X				Reach									X			X	X	X
															X			X	X
	X				Adoption									X			X	X	X
																X		X	
	X				Implementation									X			X	X	X
																X		X	X
	X				Appropriateness											X		X	X
	X				Feasibility										X			X	X
	X				Maintenance									X			X	X	X
												X	X						
		X	Costs	Employer, Societal	Time spent by employers and employees on activities related to MENTUPP									X			X	X	X
												X	X						
		X		Employer, Societal	Time spent by employers and employees on the MENTUPP Hub										X			X	X
		X		Employer, Societal	Absenteeism of employees due to mental health issues							X	X				X	X	X
		X		Employer, Societal	Presenteeism of employees due to mental health issues							X	X				X	X	X
		X		Healthcare	Use of primary healthcare due to physical health issues							X	X				X	X	X
		X		Healthcare	Use of primary healthcare due to mental health issues							X	X				X	X	X
		X		Societal	Time investment of ROs to implement MENTUPP									X			X	X	X
		X		Societal	Travel costs of ROs to implement MENTUPP									X			X	X	X

The outcome evaluation will examine the effectiveness of the MENTUPP intervention to generate the expected proximate, intermediate, and long-term outcomes. For the economic evaluation, a range of costs will be measured in addition to the proximate, intermediate and long-term outcomes. Participants will be invited to complete the six validated questionnaires and bespoke survey measures at three time intervals: baseline, 6-month follow-up and 9-month follow-up.

### Outcome evaluation

Six validated instruments consisting together of 61 items for the outcome evaluation will be administered to participants:

- Mental wellbeing and quality of life: The World Health Organisation – Five Wellbeing Index (WHO-5) [67]
- Depression: Patient Health Questionnaire (PHQ-9) [68]
- Anxiety: Generalized Anxiety Disorder Scale (GAD-7) [68]
- Depression Stigma: Depression Stigma Scale (DSS) [69]
- Burnout: Burnout Assessment Tool (BAT) [70]
- Presence (level) of psychosocial risk factors, work resources and stressors: 19 items selected from the Copenhagen Psychosocial Questionnaire, Version III (COPSOQ-III) [71]

Two bespoke surveys, a pre- and post-intervention survey, will be used to measure various aspects related to the outcome, process and economic evaluation of the MENTUPP intervention. The pre-intervention survey will be conducted with all participants at baseline and consists of 34 items: sociodemographic and work-related information, experience with mental health difficulties, initial expectations and intentions with regard to MENTUPP, presenteeism and absenteeism, healthcare and mental healthcare use, and proximate and intermediate outcomes of the MENTUPP intervention. The post-intervention survey is conducted with all participants at 6-month and 9-month follow-up and consists of 28 items and overlaps largely with the pre-intervention survey. Data are collected related to the following themes: experience with the MENTUPP Hub, experience with mental health difficulties, presenteeism and absenteeism, healthcare and mental healthcare use, and proximate and intermediate outcomes of the MENTUPP intervention (see Tables 1 and 2 for more information on the measures used).

**Table 2:** Overview of evaluation measures and instruments

Type of measure	Measure	Measure description	Time of data collection			Type of evaluation		
			Baseline	6 months	9 months	Outcome	Process	Economic
6 validated scales	WHO-5: wellbeing [63]	The WHO-5 consists of **5 items** measuring mental wellbeing. The questionnaire uses solely positively phrased items (thus no symptom-related language), which participants have to rate on a 6-point Likert scale ranging from 0 to 5	X	X	X	X		X
	PHQ-9: depression [64]	The PHQ-9 consists of **9 items** that measure symptoms of depression over the last two weeks. The items need to be rated on a 4-point Likert scale (i.e. not at all – several days – more than half the days – nearly every day)	X	X	X	X		X
	GAD-7: anxiety [64]	The GAD-7 is a **7-item** scale that measures the presence of anxiety-related problems over the last two weeks. The items need to be rated on a 4-point Likert scale (i.e. not at all – several days – more than half the days – nearly every day). An eighth item assesses the level of difficulty with certain aspects of life due to anxiety-related problems and ranges from 0 (i.e. not difficult at all) to 3 (i.e. extremely difficult)	X	X	X	X		X
	DSS: personal stigma [65]	The personal stigma subscale of The DSS consists of **9 items** and measures respondents’ personal attitudes towards depression. As for the pilot, the items are slightly reformulated and measure attitudes towards depression and anxiety, instead of only attitudes towards depression. This is a common practice and does not affect the validity or reliability of the scale. The items need to be rated on 5-point Likert scale	X	X	X	X		X
	BAT: burnout [66]	The BAT consists of **12 items** and measures 4 dimensions of burnout: exhaustion, mental distance, emotional impairment, cognitive impairment. Contrary to the Maslach Burnout inventory it measures cognitive malfunctioning and produces a single burnout score that is easy to interpret. The items need to be rated on a 5-point Likert scale	X	X	X	X		X
	COPSOQ-III: psychosocial factors in the workplace [67]	Ten psychosocial work environment dimensions^a^ plus three higher-level constructs^b^ measured with a total of **19 items**, are selected to measure the psychosocial environment in MENTUPP. The items are derived from the Copenhagen Psychosocial Questionnaire, version III (COPSOQ-III)	X	X	X	X		X
2 bespoke surveys	Pre-intervention bespoke survey	Two bespoke surveys, a pre- and post-intervention survey, will be used to measure various aspects related to the outcome, process, and economic evaluation of the MENTUPP intervention. The bespoke survey at baseline consists of 34 items	X			X	X	X
	Post-intervention bespoke survey	The bespoke survey completed at 6-month and 9-month follow-up consists of 28 items and overlaps largely with the baseline bespoke survey		X	X	X	X	X
Monitoring instrument	Characteristics of the SME and implementation	A monitoring instrument developed by the MENTUPP consortium will be used to collect information from each SME in close consultation with the main contact person or designated ‘Champion’ to track relevant information about the organisation, the implementation of MENTUPP within the organisation, and cost-related outcomes	X	X	X		X	X
User data	User information on participants’ use of the Hub	Participant User data will be collected within the MENTUPP Hub to provide the frequency (total number) of registration, logins, and visits to pages and intensity (length of time) for the various pages and overall time spent in the Hub		X	X		X	X
Focus group	With ROs	Focus groups will be carried out with the ROs to collect additional information on the implementation process of MENTUPP		X	X		X	

^*^1. Quantitative demands at work (2 items), 2. Work pace (1 item), 3. Influence at work (4 items), 4. Recognition (1 item), 5. Quality of leadership (4 items), 6. Mutual trust between employees (1 items), 7. Trust regarding management (2 items), 8. Justice at work (2 items), 9. Social support from colleagues (1 item), 10. Social support from supervisors (1 item)

^**^Combinations of selected items from the dimensions above will be used to approximate higher-level constructs of work stress (job strain and effort-reward imbalance) [6, 7] and of workplace social capital [72, 73]

### Process evaluation

The process evaluation will be conducted using a combination of the following qualitative and quantitative measures: two bespoke surveys (described above), a monitoring instrument, user data of the MENTUPP Hub users, and a focus group with the local research officers.

A monitoring instrument developed by the MENTUPP consortium will be used to collect information from each of the participating SMEs in close consultation with the main contact person or designated ‘Champion’ to inform the process evaluation. The instrument will track relevant information related to the process evaluation. Information about the organisation at the time of recruitment, including information about mental health-related activities and mental health support for employees in the organisation at the time of recruitment and after implementation of MENTUPP, will be gathered in the monitoring instrument. Furthermore, information on the implementation of MENTUPP in the organisation (events and activities that may impact implementation) and cost-related outcomes at the level of the SME will also be collected.

Pseudonymised log data of the MENTUPP Hub will be extracted at 6-month and 9-month follow-up as part of the process evaluation (feasibility and reach) to provide detail on the intensity and frequency of the use of the MENTUPP Hub online platform.

After the 6-month follow-up, focus groups with local research officers in each country will be conducted to collect more in-depth qualitative information with respect to the implementation process, appropriateness, feasibility, and maintenance of the MENTUPP Hub. During the focus group the following topics will be discussed: experiences regarding the recruitment and participation at the SME and employee level, experience with the implementation process of MENTUPP in the SMEs, country, sector specific characteristics, online format of MENTUPP and barriers and facilitators to implementation.

## Economic evaluation

For the economic evaluation, the six validated scales, the two self-developed surveys, the monitoring instrument and the log data of the MENTUPP Hub users will all be used to conduct a cost-effectiveness and cost-consequence analysis. Costs will be monitored in relation to conducting e-mental health tools and printing of leaflets and posters in all recruited SMEs. Costs in terms of productivity will be monitored via productivity/SME economic and employee turnover, absenteeism and presenteeism in the intervention and control SMEs. Absenteeism will be assessed by both self-report using customised items deduced from the iMTA Productivity Cost Questionnaire [74] and the Client Service Receipt Inventory [75]. The user data from the MENTUPP Hub will also be analysed to establish patterns of using the Hub, total number of visits and total time spent engaging with the content.

## Data management

Qualtrics [76], a General Data Protection Regulation (GDPR) compliant online platform, will be used to collect data from the validated questionnaires and the two self-developed surveys. To allow matching of the data collected of the same subject at multiple timepoints, an anonymous subject-generated identification code (ID-code) will be used [77]. For participants within the control group, the Qualtrics links will be distributed by email from the local research officers. Participants will be asked to answer four neutral questions at the first of the three time points of data collection to generate an anonymous unique ID-code and ensure confidentiality. For participants in the intervention group, the Qualtrics links will be embedded in the registration process for the MENTUPP Hub. When registering for the MENTUPP Hub, participants will be asked to enter a self-chosen 4-digit code and the SME code.

## Data analysis

### Outcome evaluation

The outcomes gathered from the six survey measures will be analysed in linear and logistic mixed effects models. The analyses will incorporate baseline and both follow-up timepoints. Changes to the outcomes will be assessed between the intervention and control groups in these models. Furthermore, descriptive analyses will be conducted on baseline characteristics and both follow-up characteristics to understand the findings from the mixed effects models. Differences between countries will be analysed in accordance with the Aspen/Indigo study [78].

### Process evaluation

Descriptive statistics will be used to analyse numeric data of the process evaluation collected via the monitoring instrument, the bespoke surveys and the user data. Qualitative data coming from the focus groups and the monitoring instrument (open text fields) will be analysed thematically. Data gathered via the focus groups with research officers will be audio recorded, transcribed, translated to English where necessary and analysed via thematic analysis. The process evaluation will be informed by the framework based on the three themes of implementation, mechanisms and context [79].

Adherence data are collected in the intervention group in three ways: via the pre-intervention survey, the post-intervention survey and the log data of the Hub. The pre-intervention survey asks participants to indicate whether they have positive expectations about the intervention at start and are intending to make use of the intervention. In the post-intervention survey, respondents are asked to indicate how much time they spent in the past months on MENTUPP-related activities. Finally, the log data of the Hub allows tracking which respondents registered for the Hub, how many times they visited the Hub, how much time they spent on the Hub and how their Hub visits are spread in time during the implementation period. Exploratory analyses will be conducted on the adherence data and results on the intention of respondents to adhere to the intervention at start, the amount of adherence during the intervention period and subsequent withdrawal from the intervention will be reported in accordance with the 2010 Consolidated Standards of Reporting Trials (CONSORT) statement.

### Economic evaluation

A cost-consequence analysis will be conducted to compare differences in costs and outcomes between the intervention and control groups to be calculated; this allows the costs of delivering and implementing MENTUPP to be compared with the different measures collected in the outcome evaluation. Multiple incremental cost-effectiveness ratios comparing costs with different outcome measures can then be determined.

Three perspectives will be used in the economic evaluation: an employer perspective, a healthcare perspective and a societal perspective. The employer perspective considers the costs of mental health issues that are borne by the employer in terms of productivity loss as indicated from data from the surveys. The costs of implementing the intervention will be calculated from the surveys, the monitoring instrument, and the log data from the MENTUPP Hub users. The healthcare perspective only includes costs that are expended on healthcare services funded by the health system and will be analysed based on usage of data from the surveys. The societal perspective includes all costs borne by the whole of society, including productivity costs or other costs not borne by the health system or the employer. This will include data from the surveys, the log data from the MENTUPP Hub, and the monitoring instrument to consider time investment and routine rather than research-related travel costs associated with implementing MENTUPP.

Data on the resources required to develop and implement MENTUPP are being collected and appropriate unit costs will be attached to time and materials used. Bespoke questionnaires have been designed to collect data from individual participants in the two arms of the trial on health service use at baseline, 6-month and 9-month follow-up periods; this will be compared with outcome data collected for these individuals over the same time periods. Data on changes in workplace productivity, including absenteeism and presenteeism are also being collected. Country-specific unit costs for healthcare service use, as well as country and industry-specific hourly wage rates, will be applied to all service utilisation and productivity impacts. To allow for multi-country comparison, final costs across countries will be converted into purchasing power parity adjusted international dollars for a common price year using the Campbell-Cochrane Economics Methods Groups EPPI-Centre Cost Converter [80].

Differences in mean costs will be compared between the two arms of the trial using bias corrected and accelerated bootstrapping. Incremental cost-effectiveness ratios (ICERs) per change in each of the six main outcomes will be calculated at 6-month and 9-month follow-up points. Statistical uncertainty will be explored through bootstrapping 5000 randomly resampled pairs of costs and each of the six outcomes and cost-effectiveness planes will then be drawn.

### Missing data

Primary statistical analyses will be run on an intention-to-treat basis, whereby all participants are included according to their group allocation and irrespective of their adherence to the intervention. Secondary analyses will be performed to account for non-adherence to the intervention. Per-protocol analyses will be run, excluding participants from the intervention group who spent hardly any time on the Hub (e.g. visited the Hub only once). In additional analyses, adherence scores will be entered either as a categorical or continuous factor in the statistical analyses (e.g. repeated measures analysis of variance) to identify whether non-adherence or low-adherence alters the outcomes in the intervention group. At follow-up, we expect quite a bit of dropout and thus missingness of data with respect to the primary and secondary outcomes. Based on inspection of the data (i.e. amount of missingness and validity of underlying assumptions), the most appropriate approach for handling missing data (e.g. complete case analysis, single imputation, model-based methods, multiple imputation) will be chosen as no additional outcome data will be collected from participants who have withdrawn.

### Ethical considerations

Ethics approval has been received from the institutional ethics committee from each of the local research officer’s institutions and the trial is registered with ISRCTN clinical trial registry (ISRCTN14104664).

### Duty of care

The duty of care protocol and standard operating procedures for local research officers established and used in the pilot study will be applied in the cRCT [53]. Contact information for international and local mental health services and supports and online links has been verified. Contact information for the local research officers has also been updated.

### Data protection

Data management and analysis will be controlled by MENTUPP consortium partners based at KU Leuven. The lead investigators of KU Leuven are responsible for developing and updating the data management plan, monitoring the implementation of the data management plan and managing the multi-country datasets. The Data Protection Officers of KU Leuven and UCC are responsible for the data security and privacy protection. KU Leuven is not involved in implementing interventions and collecting data and is only involved in accessing and analysing pseudonymised data. A Joint Data Controller Agreement was signed between members of the MENTUPP consortium to uphold their mutual roles and responsibilities. The use and transfer of participant data is covered by the EU GDPR, 2016 that ensures the protection of an individual’s privacy. Personal data will be collected as part of the informed consent process; however, the data will be stored separately from the evaluation data and will not be shared or reported outside the research team. A unique identification code will be generated for each participant [77] and will be used to link the log data from the MENTUPP Hub and the evaluation data across the time points and allow for participants to access, correct or withdraw any data at any time. The merged dataset will not include personal names, email addresses, mobile phone numbers or any identifiable information. Data will be transferred and stored following the principles of the EC Directive on personal data protection and confidentiality, GDPR (EC/2016/679).

### Dissemination of findings

The data collected during this trial will be analysed and reported in peer-reviewed publications and at conferences for academic and experts in the area of occupational psychology, workplace mental health and implementation science, and in webinars/blogposts for the public. Peer-reviewed publications will be prepared in line with the MENTUPP Publication Policy. Where possible, feedback will be provided to each SME based on the findings relevant to their own organisation, country and sector, provided there is an adequate sample size. The findings will also be reported back to the funding agency.

## Discussion

The MENTUPP intervention employs a multi-level approach targeting employees and leaders within an organisation and the organisation as a whole. The materials presented through the MENTUPP Hub are designed to support SMEs in promoting mental health in the workplace for the construction, healthcare and ICT sectors, which have been associated with significant and specific workplace stressors [81–83]. Although multi-level approaches are often recommended, high-quality studies testing multi-level approaches at the workplace are largely missing [22, 84, 85], with even fewer studies focusing specifically on SMEs and on the broad spectrum of mental health concerns from non-clinical mental health difficulties to clinical mental disorders. The MENTUPP intervention has been developed based on theoretical frameworks [48, 84], evidenced by recent literature [62–64], an expert questionnaire [13] and was tested in a pilot study [53].

The results of this study will provide a comprehensive overview of the implementation of such an intervention in eight European countries and Australia. The MENTUPP project will not only provide an evidence-informed, tailored intervention for employees and employers of SMEs but will also provide a thorough evaluation regarding implementation processes, effects and economic impact and thereby, provide results about mental health outcomes, the associated costs and benefits, as well as a better understanding of the barriers and facilitators to using these tools in real-life settings across the healthcare, construction and ICT sectors.

This study has several strengths. Firstly, a pilot study was conducted in the same countries prior to conducting the cRCT, and thus the implementation and evaluation of the intervention has been ecologically validated. The MENTUPP intervention, as well as the implementation and evaluation strategies, has been optimised based on a systematic review and discussion among Consortium members and external stakeholders of the experience of conducting the pilot study. The visual prompts within the intervention have been enhanced and the intervention content has been refined based on feedback received during the pilot study. The implementation process has been improved by simplifying the registration procedure, which was identified as a significant barrier for participation during the pilot study. Furthermore, the evaluation strategy has been refined to reflect the core research questions and reduce the number of questions, which will improve participant experience. Moreover, a comprehensive ToC has been developed that describes in detail how we assume the intervention to work, under which conditions and what kind of changes we expect to see on the different organisational levels in the short, intermediate and long term [66].

The present study will contribute to the advancement of workplace mental health intervention programmes by designing an evidence-based and pilot-tested intervention based on implementation facilitators identified in the pilot study. This resulted in an optimised intervention and implementation strategy that adopts a flexible approach to the implementation of the intervention, which has been shown to be a facilitator to successful implementation in the literature [55, 86]. This approach involves following a standardised method whereby the local research officer in each intervention country completes a series of checklists with the Champion in the SME to determine the best approach to implementing the intervention within their organisation. The flexible but structured approach to implementing the intervention increases the ecological validity and the representativeness of the results. The study also employs a rigorous monitoring tool to control for any confounding factors that may occur in any of the organisations.

Approximately 50% of an individual’s waking hours are spent working and this can be much more for some sectors, namely the construction, healthcare and ICT sectors [87–89]. This suggests that the workplace is a promising context for delivering mental health interventions, particularly given the literature that indicates the workplace can be associated with specific risk factors (e.g. high workload, shift work) and protective factors (e.g. peer support, supportive management) for mental health [90–92]. This study will contribute to the literature on interventions to mitigate risk factors and enhance protective factors in the workplace as well as providing further insight into the barriers and facilitators to implementation of a workplace intervention which is currently lacking [62]. Furthermore, the intervention targets all levels of the workplace from the individual to the organisational level, which increases the likelihood of its success [93]. The MENTUPP intervention addresses not only the individual, but also encouraging active participation at the management level and the whole organisation, the MENTUPP intervention encourages workplaces to be more active in primary prevention by creating mentally healthy workplaces through reduction of known risk factors and promotion of positive aspects at work.

The intervention is available in eleven languages at present to ensure delivery to individuals in a language that most people are familiar with. This increases the likelihood that the intervention will be available to all employees of an organisation in their preferred language. Access to content in their preferred language also allows employees to relate to the intervention material and contributes to reducing stigma that can often be present in relation to help-seeking and support. This is particularly useful for employees in the construction sector who are known to be a mobile workforce. Finally, the evaluation strategy involves the use of well-standardised, internationally used measures of mental health and wellbeing and psychosocial workplace risks. The various measures will provide an accurate assessment of the outcomes experienced by participants and within organisations.

Some of the limitations associated with the study relate to the current challenging times, including the COVID-19 pandemic (particularly for the healthcare sector), increase in the cost of energy and the crisis in Ukraine (particularly for bordering European countries). These challenges may inhibit an organisation’s ability to opt into and adhere to the intervention and are outside the researchers’ control. However, the research team has attempted to alleviate these challenges by facilitating access to the intervention in a self-paced manner for workers to access in their own time, by presenting relatable material on the pandemic and tips on how to cope during the pandemic. The intervention has also been translated to the Ukrainian language to accommodate workers who may have been displaced due to the war.

The participation of SMEs and participants within SMEs is voluntary, which may cause self-selection bias to the study whereby SMEs and participants who already have an inherent interest in mental health may choose to participate. Self-selection bias into the study may limit the external validity of the study and limit the generalisability of results. However, very few research projects about mental health have been conducted in the construction industry. Therefore, even if the more interested workplaces select themselves into the study, it will be an improvement to document experiences from this sector which will help with future initiatives. Furthermore, the extensive process evaluation will nevertheless give us rich knowledge about these workplaces that will help to understand what is needed to also reach less motivated workplaces. Self-report measures employed to capture any potential change to mental health outcomes may also be influenced by response bias. As outlined above, the flexible approach to the trial may result in some variability in the reach and the implementation of the intervention in SMEs. However, this flexibility is an integral part of the study and considered a strength when applying interventions in real-life conditions.

## Conclusions

The MENTUPP intervention is a multi-level, tailored, flexible and accessible workplace-based intervention designed to support SMEs in promoting and protecting mental health and wellbeing in the workplace, specifically for the construction, healthcare and ICT sectors, which have been associated with significant and specific workplace stressors. The results of this study will provide insight into the effectiveness, as well as the process and economic evaluation of the MENTUPP intervention in nine countries.

### Supplementary Information

**Additional file 1.**

**Additional file 2.**

## Data Availability

Anonymised data required to support the protocol and in accordance with the General Data Protection Regulation (2016) as well as the statistical code, can be supplied on request.

## Abbreviations

MENTUPP: Mental health promotion and intervention in occupational settings
SMEs: Small and medium-sized enterprises
cRCT: Cluster randomised controlled trial
ICT: Information and communication technology
ToC: Theory of change
NACE: Nomenclature of Economic Activities
WHO-5: The World Health Organisation – Five Wellbeing Index
PHQ-9: Patient Health Questionnaire
GAD-7: Generalized Anxiety Disorder Scale
DSS: Depression Stigma Scale
BAT: Burnout Assessment Tool
COPSOQ-III: Copenhagen Psychosocial Questionnaire, Version III
GDPR: General Data Protection Regulation
ID-code: Identification code
CONSORT: Consolidated Standards of Reporting Trials
ICERs: Incremental cost-effectiveness ratios
TSC: Trial steering committee
