# Supplementary material for: Implementation and evaluation of a multi-level mental health promotion intervention for the workplace (MENTUPP): study protocol for a cluster randomised controlled trial
Source: Trials. 2023 Sep 30;24:621. doi: 10.1186/s13063-023-07537-0 (PMC10543326; doi:10.1186/s13063-023-07537-0)
Supplement: Supplementary file 1 — Additional file 1. [file 13063_2023_7537_MOESM1_ESM.docx]

**Appendix A: Informed Consent Form**


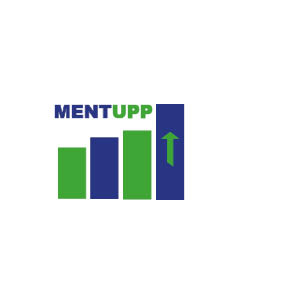


**Mental Health Promotion and Intervention in Occupational Settings: Research Project**

**Informed Consent Form**

Thank you for expressing a wish to take part in the MENTUPP study. The purpose of this form is to confirm that you agree to take part in this project and that you know what the project and your participation involves.

Please read the attached information sheet, followed by the points below, and if you are still happy to participate, please click ‘Yes, I agree’ below.

For further information about the MENTUPP Hub, please refer to the MENTUPP Hub Information and Terms of Use and the MENTUPP Hub Data Privacy Statement.

- I confirm that I have received a copy, read, and understood the information sheet and the data protection notice for this study, and have had the opportunity to ask questions.
- I am over 18 years of age.
- I understand that my participation is voluntary and that I am free to withdraw or cancel my registration to the MENTUPP Hub at any time, without giving a reason.
- I understand that participation in this research is not expected to have any negative effects on my mental health. However, should participating in this research have a negative impact on my mental health I am advised to seek support from a GP/medical practitioner and will highlight this with the project Research Officers (name and email provided in each country) or the Principal Investigator.
- If I participate in a focus group, I understand that I will be recorded and that anonymised quotes may be used in any future publications.
- I understand that the findings of this research will be shared with the study partners and may be published in academic settings as well as among the wider community but that my identity will remain anonymous.
- I understand that the MENTUPP Project is evaluating how users engage with and use interventions (e-learning tools) in the MENTUPP Hub.
- I understand that data (non-personal) generated by the MENTUPP Hub will be used to evaluate the interventions for research purposes.
- I understand that data generated will be encrypted on electronic data devices and that only anonymised evaluation results may be published.
- I understand that I can object to any further use of my data collected in the MENTUPP Hub, and that I can request that it and my personal data can be deleted.
- I agree to receive automated e-mails that are related to the use of the MENTUPP Hub.
- I understand that if I have further questions about the MENTUPP Hub, I can email (contact email provided)

**Contact details:**

Name (BLOCK CAPITALS) _________________________________

E-Mail: _______________________________________________

Participant Signature: ______________­__ Date: ______________

If you have any queries in relation to this study please contact Research Officers (name and email provided within each country).

Do you consent to participate in the study?

Yes

No
